# Anesthesia management of a patient with severe post-rheumatic mitral stenosis undergoing cesarean section

**DOI:** 10.1007/s00101-024-01469-3

**Published:** 2024-10-10

**Authors:** Stanislaw Vander Zwaag, Johan Winata, Cahit Birdir, Barbara Seipolt, Stephan Haussig, Jens Fassl

**Affiliations:** 1 Department of Cardiac Anesthesiology, Heart Center Dresden, Technical University Dresden, Fetscherstraße 76, 01307 Dresden, Germany; 2 Department of Obstetrics and Gynecology, Carl Gustav Carus University Hospital, Technical University Dresden, Fetscherstraße 74, 01307 Dresden, Germany; 3 Department of Pediatric Intensive Care, Carl Gustav Carus University Hospital, Technical University Dresden, Fetscherstraße 74, 01307 Dresden, Germany; 4 Department of Cardiology, Heart Center Dresden, Technical University Dresden, Fetscherstraße 76, 01307 Dresden, Germany

## Medical history

A 30-year-old female patient (gravida 3 para 2) was admitted to hospital in the 27th gestational week (GW) with progressive dyspnea during pregnancy.

## Observations

On admission the patient was suffering from severe tachycardia and a shortness of breath. The clinical examination revealed a diastolic murmur with a maximum in the fifth intercostal space in the midclavicular line.

## Diagnosis

**Fig. 1:**
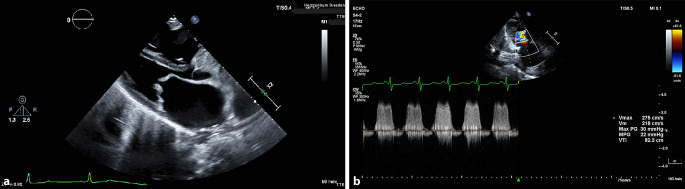
**a** Parasternal long-axis view of the mitral valve stenosis with transthoracic echocardiography. **b** Pressure measurement of the mitral valve stenosis with continuous wave (CW) doppler with transthoracic echocardiography

The computed tomography (CT) revealed a pulmonary embolism (PE) as well as a pleural effusion. Transthoracic echocardiography showed evidence of a severe mitral stenosis, with a mean pressure gradient of 22 mm Hg and an orifice area of 0.6 cm^2^. The findings are presented in Fig. 1.

## Treatment and the course

The patient was pharmacologically stabilized, including a beta-adrenergic receptor antagonist for tachycardia and heparin for PE. The case was discussed in the pregnancy heart team (cardiologists, obstetricians, neonatologists and cardiac anesthesiologists). As recommended in the ESC guidelines [1] initial stabilization and a watchful waiting until 38th GW, cesarean section (CS) with the patient under general anesthesia (GA), followed by percutaneous mitral commissurotomy (PMC) 2 weeks later.

An elective cesarean section was scheduled on 38 + 2 GW. For the surgery, we formulated the following plan:

Plan A. Beta-adrenergic receptor antagonist (bisoprolol 1.25 mg) continued on the day of surgery. Induction of GA with fentanyl, thiopentone and rocuronium, maintenance with sevoflurane up to 0.6 MAC (minimum alveolar concentration). Avoidance of maternal tachycardia, maintenance of preload and afterload, with continuous cardiac output measurement with pulse contour analysis (FloTrac and HemoSphere, Edwards Lifesciences, Irvine, CA, USA).

Plan B. In the case of hemodynamic instability, rapid initiation of extracorporeal life support with or without PMC.

The patient arrived in our hybrid operating room hemodynamically stable, in normal sinus rhythm, with heart rate (HR) 55 bpm and blood pressure (BP) 105/69 mm Hg. According to the formulated plan, we inserted the necessary catheters with the patient under local anesthesia (radial arterial line and central venous catheter, introducer sheaths for rapid initiation of veno-arterial extracorporal membrane oxygenation (VA-ECMO), as well as an introducer sheath for PMC). We started an infusion of norepinephrine, titrated to the target of normal MAP and a high-normal systemic vascular resistance (SVR) to compensate for the reduction in vascular resistance and myocardial contractility after induction of GA. We induced GA and intubated the patient. The heart rate increased to 105 bpm after intubation but rapidly decreased to 60 bpm. The baby was delivered after 4 min with an initial Apgar score of 2, improving to 6 and 7 after 5 and 10 min, respectively.

**Fig. 2:**
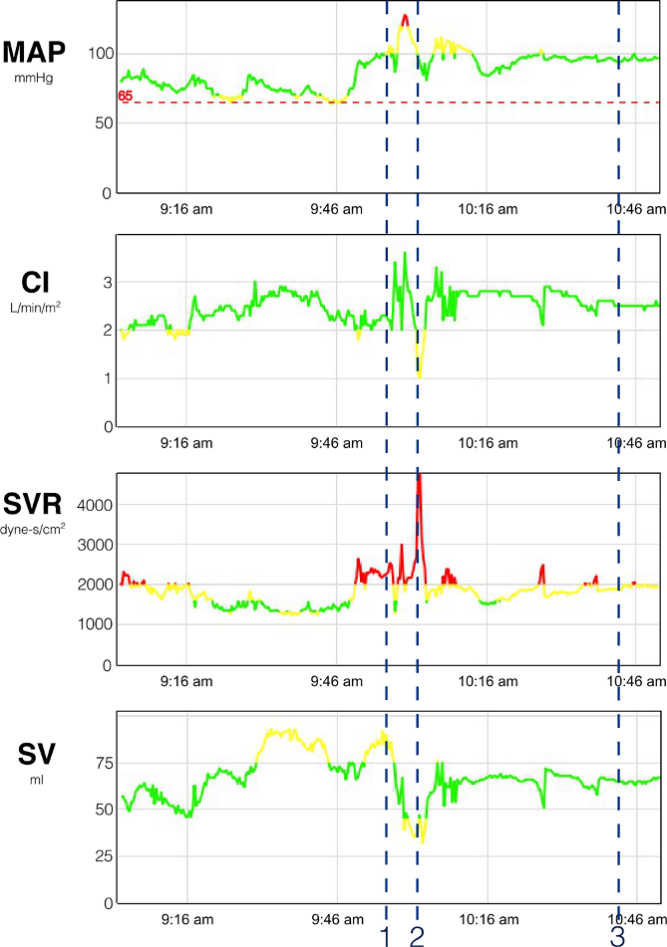
**a** Time course of the mean arterial pressure (MAP) in mmHg, *green* above the lower limit of 65 mmHg, *yellow line* close to the lower limit of the MAP or higher than the upper limit of the MAP, *red* above the upper limit of the MAP. **b** Time course of the cardiac index (CI) in l · min^-1^ · m^-2^. *Green line* above the lower limit of 2 l · min^-1^ · m^-2^. The *yellow line* indicates that the CI is under lower limit of 2 l · min^-1^ · m^-2^. **c** Time course of the systemic vascular resistance (SVR) in dyne-s/cm^2^. *Green* coded normal SVR between 1000 and 1500 dyne-s/cm^2^. The *yellow* and the *red lines* indicating a higher or very high SVR above 1500 dyne-s/cm^2^. **d** Time course of the stroke volume (SV) in ml. *Green* normal SV between 45 and 75 ml. The *yellow line* indicates an high normal or low normal SV. *Blue dashed lines* indicate the relevant events: 1 – induction of general anesthesia and surgical incision, 2 – administration of 100 µg carbetocin, 3 – end of surgery

Although the patient remained hemodynamically stable, the infusion rates of norepinephrine were higher than expected, ranging from 0.07–0.35 µg · kg^−1^ · min^−1^. The hemodynamic parameters over the course of anesthesia are presented in Fig. 2.

After completion of the surgery, we assessed the volume status of the patient in transthoracic echocardiography, downtitrated the norepinephrine infusion, and extubated the patient without complications. There was no peripartum bleeding. The patient was monitored in the intensive care unit (ICU) to exclude decompensation after volume shift from a contracting uterus and was discharged on the day following the uneventful PMC 2 weeks later.

## Discussion

Safe delivery in patients with severe postrheumatic mitral stenosis and changing physiology during pregnancy remains a challenge, with a reported maternal mortality of up to 5% [2].

The decision whether to perform a PMC in a pregnant patient, should be individualized. Even though it is an effective and generally accepted treatment in the 2nd trimester [3], in patients diagnosed in the 3rd trimester the risks of inducing a preterm delivery may outweigh the benefits. We therefore decided against PMC before delivery and for the watchful waiting strategy.

Regional anesthetic techniques, e.g., spinal or epidural anesthesia, might be advantageous in patients with mitral stenosis (MS). Epidural anesthesia enables titration of doses to maintain hemodynamic stability [4]; however, the language barrier in our patient excluded the possibility to safely provide regional anesthesia. With GA, administration of beta-adrenergic receptor antagonists and a sufficient dose of opioid enable intubation without reflex tachycardia. Esmolol, with its short half-life, is an attractive option but it can induce fetal bradycardia [5]. Although we had esmolol ready to use, the morning dose of beta-adrenolytic sufficed to maintain a normal heart rate.

There are few data regarding the use of extracorporeal life support (ECLS) in the peripartum or postpartum period. Fayad et al. reported providing hemodynamic support to a decompensated patient with severe postrheumatic mitral stenosis. In their case, however, the cesarean section was an emergency, and the patient underwent open valve surgery. The ECMO enabled the initial stabilization of the patient [6]. Anticipating the risk of pulmonary edema or heart failure, we decided to insert the vascular accesses prior to the induction of GA to be able to immediately initiate ECLS.

In conclusion, with the involvement of a multidisciplinary pregnancy heart team, cesarean delivery in GA can be safely performed. Anticipating the physiological changes and developing a rescue plan are the cornerstones of patient safety in these difficult cases.

## Declarations

For this article no studies with human participants or animals were performed by any of the authors. All studies mentioned were in accordance with the ethical standards indicated in each case. For images or other information within the manuscript which identify the patient, consent was obtained.
